# Immune system stimulation by the native gut microbiota of honey bees

**DOI:** 10.1098/rsos.170003

**Published:** 2017-02-08

**Authors:** Waldan K. Kwong, Amanda L. Mancenido, Nancy A. Moran

**Affiliations:** 1 Department of Integrative Biology, University of Texas at Austin, Austin, TX, USA; 2 Department of Ecology and Evolutionary Biology, Yale University, New Haven, CT, USA

**Keywords:** antimicrobial peptides, apidaecin, innate immunity, symbiosis

## Abstract

Gut microbial communities can greatly affect host health by modulating the host's immune system. For many important insects, however, the relationship between the gut microbiota and immune function remains poorly understood. Here, we test whether the gut microbial symbionts of the honey bee can induce expression of antimicrobial peptides (AMPs), a crucial component of insect innate immunity. We find that bees up-regulate gene expression of the AMPs apidaecin and hymenoptaecin in gut tissue when the microbiota is present. Using targeted proteomics, we detected apidaecin in both the gut lumen and the haemolymph; higher apidaecin concentrations were found in bees harbouring the normal gut microbiota than in bees lacking gut microbiota. In *in vitro* assays, cultured strains of the microbiota showed variable susceptibility to honey bee AMPs, although many seem to possess elevated resistance compared to *Escherichia coli*. In some trials, colonization by normal gut symbionts resulted in improved survivorship following injection with *E. coli*. Our results show that the native, non-pathogenic gut flora induces immune responses in the bee host. Such responses might be a host mechanism to regulate the microbiota, and could potentially benefit host health by priming the immune system against future pathogenic infections.

## 1. Introduction

Bacterial communities living in symbiosis with animal hosts can be important factors in host health. For instance, many insects harbour beneficial gut microbiotas that aid in food digestion and disease resistance [1]. The recently characterized gut microbiota of the Western honey bee, *Apis mellifera*, comprises approximately nine socially transmitted bacterial species that probably have long evolutionary associations with their host [2–5]. This distinctive, specialized gut community is likely to affect host physiology, but the interactions between bees and their resident microbes are still largely uncharacterized.

One route through which the gut microbiota might influence bee health is by the modulation of host immune responses. Antimicrobial peptides (AMPs) are a key component in insect innate immunity for defence against invading pathogens. These short peptides are released during bacterial, fungal and protozoan infection, and damage microbial cells by perforating membranes and inhibiting protein folding [6]. Four families of AMPs (abaecin, apidaecin, defensin and hymenoptaecin) are induced in the honey bee haemolymph upon immune challenge by Gram-negative and Gram-positive bacteria [6–9].

In this study, we investigate the influence of the honey bee's native gut bacteria on host immune function. We examine: (i) if the presence of the gut microbiota affects gene expression of AMPs; (ii) whether there are corresponding shifts in peptide concentrations in the gut lumen and haemolymph; (iii) if member species of the gut microbiota are resistant to bee AMPs and (iv) if the gut microbiota is beneficial to host survival after wounding and bacterial infection.

## 2. Material and methods

Bees used in this study originated from *A. mellifera* colonies kept at the University of Texas at Austin. To acquire uninoculated bees (bees lacking normal microbiota and containing little or no other bacteria), *A. mellifera* pupae were aseptically removed from their cells and raised in sterile cages until inoculation. Newly eclosed bees were hand-fed 5 µl of (i) approximately 10^6^ bacterial cells of *Snodgrassella alvi* wkB2 [3] or *Gilliamella apicola* wkB7 [10] suspended in 250 µl of sucrose water, (ii) whole guts from bees homogenized in 250 µl sucrose water or (iii) a sterile sucrose water control. Bees were then transferred to plastic cup cages and kept at 34°C for 5 days and supplied with filter-sterilized 1 : 1 (w/v) sucrose water and sterile pollen. Successful creation of gnotobiotic bees with cultured *S. alvi* or of mixed communities from whole-gut contents have been previously shown, and control (uninoculated) bees have been found to consistently contain less than 10^5^ bacterial cells [11–14]. Assays to assess *G. apicola* colonization are described in the electronic supplementary material.

### 2.1. Antimicrobial peptide gene expression

**Figure 1.**
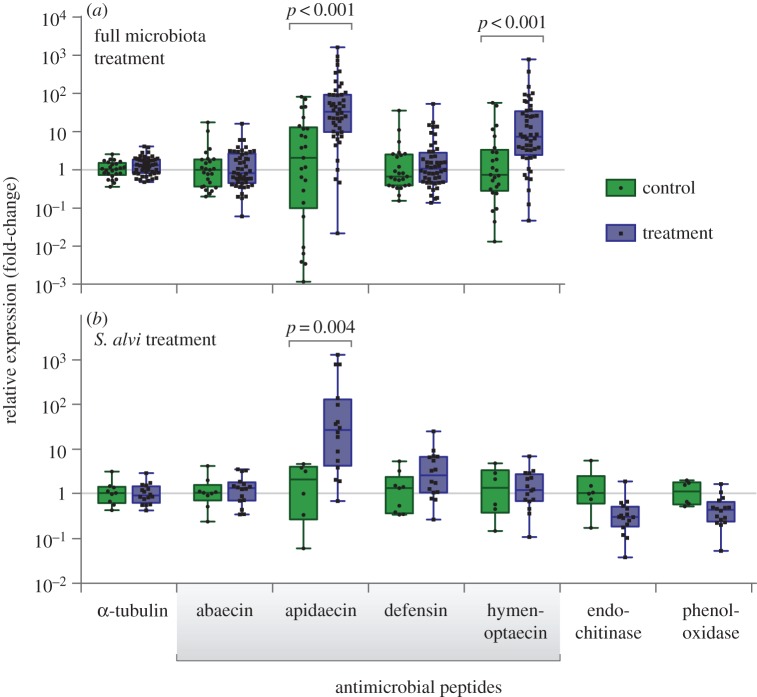
The honey bee gut microbiota up-regulates host expression of AMPs. Two experiments are shown: (*a*) bees inoculated with guts of hive bees (treatment) or guts of newly emerged bees lacking microbiota (control); (*b*) bees inoculated with cultured *S. alvi* strain wkB2 (treatment), or sterile sucrose solution (control). Relative expression values indicate fold-change compared to the mean value of control.

Whole guts from each bee were removed for RNA extraction (TRIzol protocol, Thermo Fisher Scientific Inc.; see the electronic supplementary material) and cDNA synthesis (Verso cDNA synthesis kit, Thermo Fisher Scientific Inc.). Relative gene expression was determined using quantitative PCR (qPCR). The fold-change in expression between uninoculated and inoculated bees was determined for the genes encoding α-tubulin (primers from [15]), abaecin [16], apidaecin [15], defensin [17], hymenoptaecin [18], endochitinase [19] and phenoloxidase [17]. Ten-microlitre qPCR reactions were carried out on an Eppendorf Mastercycler ep realplex or a Thermo Fisher ViiA7 using iTaq Universal SYBR Green Supermix (Bio-Rad Inc.). Expression levels were measured in triplicate for each biological replicate and normalized against the housekeeping gene RPS5 [16]. Statistical analyses were performed in Prism 6 (GraphPad Software Inc.) using two-tailed *t*-tests with the Holm–Šídák correction for multiple comparisons. Tests of significance in figure 1 were performed on untransformed qPCR cycle threshold (*C*_T_) values.

For the gut-fed qPCR experiment, two cohorts of the full microbiota treatment were used; bees of each cohort were fed gut material from a single adult hive bee. In the control treatment, bees were fed gut material from a newly emerged, uninoculated adult worker bee. After 5 days, DNA and RNA were extracted from the whole guts of a subset of control and treatment bees. 16S rDNA amplicons were sequenced to assess the composition of the gut microbial community of these bees (see the electronic supplementary material).

### 2.2. Detection of apidaecin *in vivo*

Newly eclosed bees were fed guts of hive bees or of newly emerged, uninoculated bees and kept at 34°C with sterile 1 : 1 (w/v) sucrose water, without pollen. After 5 days, haemolymph from the thorax and gut fluid from the rectal lumen were withdrawn using micropipettes after puncture by glass needles. Samples were prepared for reverse-phase liquid chromatography--tandem mass spectrometry (LC-MS/MS) using the peptide extraction and enrichment protocol adapted from [19]. Haemolymph and gut fluid samples were pooled from 5 to 13 bees to obtain sufficient concentrations for analysis. LC-MS/MS was conducted by the University of Texas Proteomics Facility on a Dionex Ultimate 3000 Nano UPLC instrument coupled to an Orbitrap Elite mass spectrometer (Thermo Fisher Scientific Inc.). Detailed protocols are described in the electronic supplementary material.

### 2.3. Antimicrobial peptide resistance assays

To optimize the activity of AMPs and growth of bacteria, growth inhibition assays in liquid media were performed using 50% diluted brain heart infusion broth (BHI) or 50% Lactobacillus MRS media (BD Difco). AMPs (synthesized by NovoPro Bioscience Inc.) were serially diluted twofold in 96-well culture plates to obtain concentrations ranging from 0.78 to 100 µg ml^−1^. Overnight bacterial cultures from blood heart infusion agar plates (Hardy Diagnostics) or Columbia agar plates (BD Difco) were diluted to an optical density (OD_600_) of 0.5 in 50% BHI or 50% MRS, diluted 1 : 100, and distributed to wells for a total volume of 200 µl. Plates were incubated at 35°C, 5% CO_2_. Optical density data were recorded for 2 days and used to generate growth curves. Minimum inhibitory concentration (MIC) was defined as the lowest concentration of AMP that notably inhibited growth of bacteria compared to controls.

For inhibition zone assays, overnight bacterial cultures were swabbed onto twofold diluted blood heart infusion agar, and 6 mm filter paper discs containing 5 or 10 µg of apidaecin Ia and Ib were placed on the surface of the agar. Plates were incubated at 35°C in 5% CO_2_ overnight and then observed for zones of inhibition.

### 2.4. Bacterial clearing and survival experiments

To measure the immune response of bees after bacterial challenge, we injected 1 µl of *E. coli* ATCC 25922 (10^3^–10^5^ cells µl^−1^) into the abdomen of uninoculated and inoculated bees using a fine-tipped glass capillary. At 2 and 6 h post-injection, haemolymph was collected from each bee and 0.5 µl aliquots of undiluted and 1 : 100 diluted haemolymph were plated onto tryptic soy agar. After overnight incubation at 37°C, the number of colony-forming units (CFUs) per agar plate was counted to calculate the number of *E. coli* cells per microlitre of haemolymph. Bees not used for haemolymph extraction were monitored for survival, and the number of deceased bees for each treatment group was recorded approximately every 3 h.

## 3. Results

When compared to bees lacking gut microbiota, bees inoculated with the normal gut microbiota via feeding with hive bee guts or with the gut symbiont *S. alvi* [3] had significant increases in transcripts for apidaecin (figure 1), with an average 28.5-fold increase in the full microbiota-fed bees and an average 26.9-fold increase in *S. alvi*-fed bees in gut tissue. In the bees inoculated with guts of hive bees, upregulation of hymenoptaecin was also observed, with an average 8.1-fold increase in transcript abundance in gut tissue. Expression levels for abaecin and defensin did not show significant differences between the control and treatment groups, nor were there differences in the expression of the other examined genes in gut tissue. 16S rDNA-based community analysis verified that bees fed hive bee guts were successfully colonized with the characteristic microbiota consisting of *S. alvi*, *Gilliamella apicola*, *Frischella perrara*, *Bifidobacterium* spp., and *Lactobacillus* Firm-4 and Firm-5 (electronic supplementary material, figure S1).

In *S. alvi*-fed bees, we also examined expression of genes encoding phenoloxidase, which is involved in the melanization immune response [16], and endochitinase, which is up-regulated upon exposure to the pathogen *Nosema ceranae* [18]. Neither of these genes showed significant expression differences between *S. alvi*-treated bees and bees lacking gut microbiota, although they trend towards lower expression in the *S. alvi* treatment.

**Figure 2.**
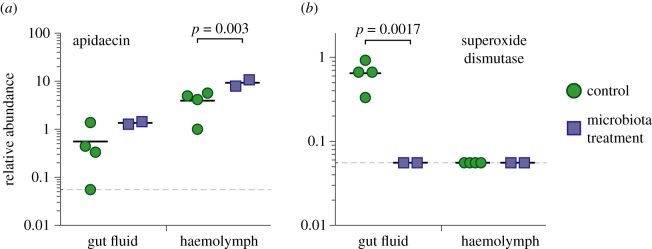
The gut microbiota alters levels of immunity-related proteins in the gut and in the haemolymph. Relative abundances are reported as peptide spectrum matches per microlitre of sample (gut fluid or haemolymph). Solid lines denote means; dashed line indicates the detection threshold.

Using a targeted LC-MS/MS approach, we determined the relative concentrations of apidaecin in haemolymph and in fluid from the gut lumen (figure 2). Apidaecin was present in both compartments, with an average 7.0-fold higher concentration in the haemolymph compared with the gut lumen. Bees colonized with the normal gut microbiota exhibited an average 2.4-fold increase in apidaecin concentration in both compartments, although the difference in the gut fluid did not reach significance. Serendipitously, our assay also detected peptides corresponding to a Cu-Zn superoxide dismutase (A0A088A933_APIME) in the gut lumen of bees lacking gut microbiota, but not in any other sampling group (figure 2). This enzyme converts toxic superoxide radicals to the less harmful H_2_O_2_, and hence may play a role in the regulation of reactive oxygen species (ROS). ROS are an important mechanism for host control of the microbiota in both insects and mammals [20,21].

Members of the normal bee gut microbiota were largely resistant to apidaecin compared with the *E. coli* control (table 1; electronic supplementary material, table S1 and figures S2–S4). *Apis mellifera* expresses two dominant apidaecin isoforms, Ia and Ib, at a ratio of approximately 1 : 20 [7]. Our LC-MS/MS assay detected only apidaecin Ib; our targeted approach may have missed apidaecin Ia, which differs from Ib by the replacement of the C-terminal leucine with an isoleucine. We found that bee microbiota strains were more resistant to apidaecin Ia than to Ib. *S. alvi* strains were usually more resistant to apidaecin Ib than strains of *G. apicola*. Gram-positive microbiota species (*Lactobacillus* Firm-5, *Bifidobacterium* sp.) were highly resistant to both apidaecin and hymenoptaecin, while the Gram-negative species, particularly *S. alvi*, were more sensitive to hymenoptaecin.

**Table 1.** AMP resistances of honey bee (*A. mellifera*) gut symbionts. Results from strains isolated from other *Apis* spp. and from bumble bees (*Bombus* spp.) are presented in the electronic supplementary material, table S1. Growth curves from MIC assays are shown in the electronic supplementary material, figures S2–S4. MIC, minimum inhibitory concentration in liquid media; IZ, inhibition zone diameter on solid media; —, no zone; n.d., not determined.

	apidaecin Ia			apidaecin Ib			hymenoptaecin
bacterial strain	MIC (µg ml^−1^)	IZ (mm) 5 µg	IZ (mm) 10 µg	MIC (µg ml^−1^)	IZ (mm) 5 µg	IZ (mm) 10 µg	MIC (µg ml^−1^)
*Snodgrassella alvi*							
wkB2	>50	—	—	12.5	—	—	3.125
wkB332	>50	—	—	25	—	—	6.25
*Gilliamella apicola*							
wkB1	12.5	9	11	3.125	12	16	>50
wkB7	3.125	—	—	1.56	—	8	n.d.
M1-2G	50	—	8	12.5	9	11	25
M6-3G	12.5	11	13	1.56	14	16	n.d.
P62G	25	7	8	3.125	11	12	>50
*Frischella perrara*							
PEB0195	n.d.	8	9	n.d.	10	12	n.d.
*Lactobacillus* Firm-5							
wkB8	>50	n.d.	n.d.	>50	n.d.	n.d.	>50
wkB10	>50	n.d.	n.d.	>50	n.d.	n.d.	>50
*Bifidobacterium* sp.							
wkB3	>50	n.d.	n.d.	>50	n.d.	n.d.	>50
*Escherichia coli*							
ATCC 25922	1.56	8	12	1.56	9	11	50

**Figure 3.**
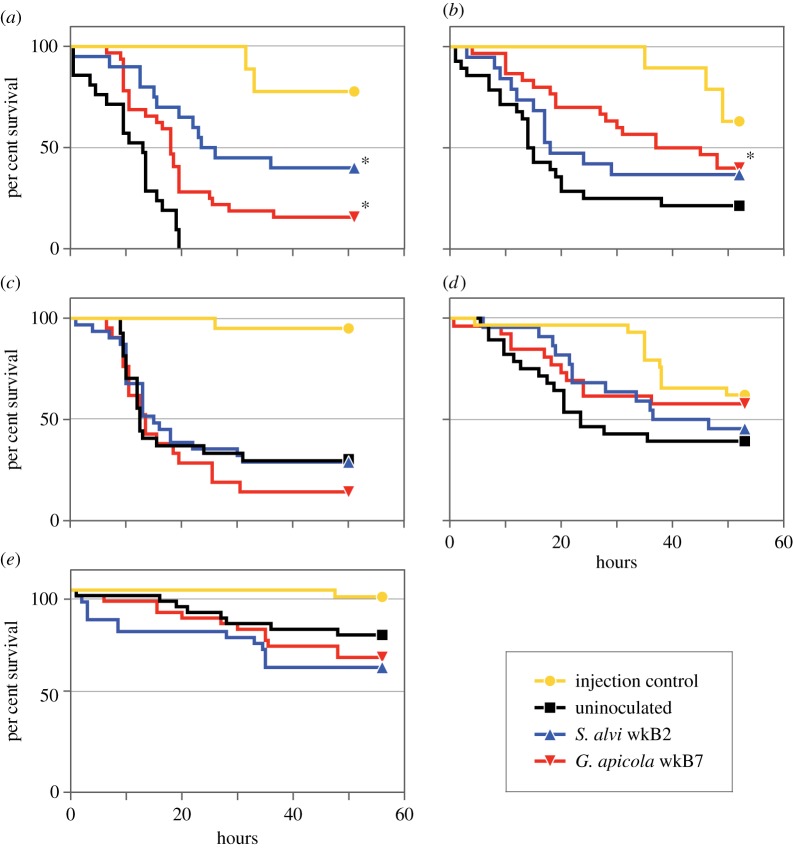
Survival rates of uninoculated and inoculated bees after *E. coli* infection. Bees were each injected with 1 µl of *E. coli* culture with 10^3^ cells (*a*,*b*), 10^4^ cells (*c*,*d*) or 10^4^ cells after 6 h of starvation (*e*). Bees inoculated with *S. alvi* wkB2 or *G. apicola* wkB7 exhibited higher survival rates after infection in two of five trials; significant differences from uninoculated treatment are denoted by asterisks. (*a*) wkB2, *p* < 0.0001; wkB7, *p* = 0.001; (*b*) wkB7, *p* = 0.0148; (*c*–*e*) results were not significant (log-rank test).

**Figure 4.**
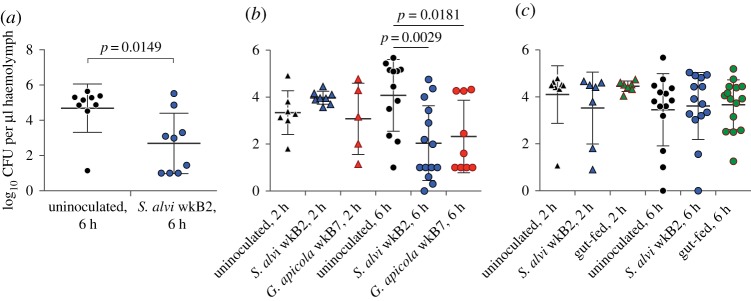
*Escherichia coli* CFUs per microlitre of haemolymph in uninoculated bees and bees inoculated with cultured *S. alvi*, *G. apicola* or crushed guts at 2 and 6 h after infection with 10^5^ *E. coli* cells. (*a*) Trial 1. Inoculated bees cleared more bacteria after *E. coli* infection, as compared to uninoculated bees (unpaired *t*-test). (*b*) Trial 2. At 6 h after *E. coli* infection, bees inoculated with *S. alvi* wkB2 and *G. apicola* wkB7 cleared more bacteria from haemolymph than uninoculated bees (unpaired *t*-test). (*c*) Trial 3. There was no difference in the amount of bacteria remaining between the control and treatment groups (unpaired *t*-test).

Results for whether the gut microbiota can facilitate the clearing of infections within the haemocoel were mixed. In two of five *E. coli*-challenge survival trials, bees that were initially inoculated with *S. alvi* wkB2 or *G. apicola* wkB7 showed significantly higher post-infection survival rates when compared with bees lacking gut symbionts (figure 3). In three trials, survivorship differences were not significant. In two of three *E. coli*-challenge clearing trials, bees inoculated with *S. alvi* wkB2 or *G. apicola* wkB7 had significantly fewer *E. coli* in their haemolymph at 6 h post-challenge compared to uninoculated bees (figure 4). In the other trial, differences were not significant.

## 4. Discussion

This study provides experimental evidence of an interaction between the honey bee gut microbiome and the immune system. We found strong upregulation of the AMP apidaecin, as well as elevated levels of hymenoptaecin, in bees inoculated with the microbiota*.* There may be differential regulation by different members of the microbiota: colonization by *S. alvi* alone did not increase expression of hymenoptaecin, suggesting that *S. alvi* does not produce the microbe-associated molecular patterns for the pathway leading to hymenoptaecin production. Hymenoptaecin is thought to be controlled by the Imd pathway, while identity of the regulatory cascade leading to apidaecin expression remains unclear [6,22]. Intriguingly, *S. alvi* appears to be more susceptible to hymenoptaecin than the other microbiota members are (table 1). In the bee gut, each species has a characteristic distribution; *S. alvi* is dominant in the ileum, while Gram-positive species are highly abundant in the rectum [2]. We examined transcription at the whole-gut level and thus could not detect any gut region-specific AMP expression patterns. Similarly, differential expression of defensins, which are primarily produced in glands of the head and thorax [23,24], could have been missed. In *Drosophila*, expression of immune genes differs strongly among gut regions [25], and the same is probably true in bees.

Most studies of honey bee AMPs have focused on their presence in the haemolymph, where they are highly up-regulated upon bacterial infection and form crucial components of the host immune response [7–9,16,26]. Our results show that microbial symbionts in the bee gut can influence the abundance of an AMP, apidaecin, in the haemolymph. This suggests that the microbiota exerts a systemic immune effect, rather than being only localized to the gut. Furthermore, we demonstrate that apidaecin occurs in the gut lumen, opening the possibility that the host immune system plays a role in structuring the gut microbial community.

A potential function of AMPs in the gut is the maintenance of microbiome homoeostasis, by selectively inhibiting foreign bacteria and by keeping native bacteria from overproliferating. Resident gut bacteria tend to have increased tolerance of host AMPs compared with allochthonous microorganisms [27], and our resistance assays suggest this is true for the bee microbiota. Although absolute concentrations of AMPs in the gut are not known, apidaecin concentrations of 7.5–18.6 µg ml^−1^ have been reported in haemolymph [19]. According to the ratio observed in our peptide data (figure 2), this would correspond to approximately 1.1–2.7 µg ml^−1^ apidaecin in the gut, a range tolerated by most bee microbiota strains (table 1). However, MIC values of AMPs should be interpreted with caution, as we found that AMP activity is highly sensitive to growth conditions, and the effectiveness of AMPs in host tissue can differ from *in vitro* results [28]. Our results were based on comparison with an *E. coli* strain commonly used in antimicrobial susceptibility assays [29]; tests using additional strains, as well as with close relatives of the bee gut symbionts, will be needed to better contextualize the magnitude of the microbiota's resistance to host AMPs.

Production of ROS is another mechanism for host control of the microbiota. In *Drosophila*, the dual oxidase (DUOX) pathway for ROS production can be induced by foreign bacteria through secretion of uracil [30]. A recent transposon mutagenesis screen in *S. alvi* identified pathways for stress response, including DNA-break repair, as important for successful bee gut colonization [13]. This implicates a role for ROS or other cytotoxic agents in modulating the microbiota within the bee gut lumen. ROS can damage both host and microbial cells, and our finding that superoxide dismutase is elevated in guts of uninoculated bees suggests that hosts neutralize ROS for self-protection in the absence of microbial targets [31].

Experiments on whether the honey bee gut microbiota confers protective benefits against pathogens and parasites have given mixed results [12,32–34]. The related gut microbiota of bumble bees decreases infection by trypanosomatid parasites [35], although this protective effect varies with microbiota composition, host genetic background and parasite strain [36,37]. The mechanisms of parasite suppression are also unclear, but given that AMPs can be potent against infectious invaders [38–40], stimulation of the immune system by the gut microbiota may contribute. We found that bees lacking their normal gut microbiota have a lower basal expression of AMPs, which could negatively affect the host's ability to fight infection (figures 1 and 2).

In our *E. coli*-challenge experiments (figures 3 and 4), we did not find a clear-cut effect of the microbiota on bee survival or the ability to clear a bacterial infection of the haemolymph. A subset of trials showed significantly improved outcomes for microbiota-colonized bees, and bees with the microbiota never performed worse than bees lacking the microbiota. This suggests that the microbiota is generally not harmful and, in some cases, can be helpful in warding off pathogens. The observed variation has several potential explanations, as the experimental trials were performed at different seasons using different cohorts of bees; for example, conditions during larval development potentially impact immune responses and susceptibility to pathogens.

This variation in outcomes will be better understood through elucidation of the molecular mechanisms by which the bee gut microbiota affects host immunity. Based on our results, future studies of bee immunity should take into account the gut microbiota, as variation in the immune responsiveness correlated with age or environment could also be due to differences in the microbiota. Gut microbes play an important part in modulating immunity and influencing host heath in many animal species [20,41], and the honey bee offers a valuable perspective on host–microbe interactions in the context of a social insect.

## Supplementary Material

Supplementary Materials. Supplementary Methods, Supplementary Results, five Supplementary Figures and one Supplementary Table.

## Supplementary Material

Supplementary Data Set. Gene expression (qPCR) and protein abundance data.

## Data accessibility

Data from qPCR and peptide analysis are placed in Supplementary Dataset 1. 16S rDNA sequence reads are deposited under NCBI Bioproject PRJNA345381.
