# Supplementary material for: Immune system stimulation by the native gut microbiota of honey bees
Source: R Soc Open Sci. 2017 Feb 8;4(2):170003. doi: 10.1098/rsos.170003 (PMC5367273; doi:10.1098/rsos.170003)
Supplement: Supplementary Materials. Supplementary Methods, Supplementary Results, five Supplementary Figures and one Supplementary Table. [file rsos170003supp1.pdf]

Supplementary Material for

Immune system stimulation by the native gut microbiota of honey bees
Waldan K. Kwong, Amanda L. Mancenido, Nancy A. Moran
in
Royal Society Open Science

Contents:
Supplementary Methods
Supplementary Results
Supplementary References
Figures S1 to S5
Table S1

Supplementary Methods

Bacterial cultures

S. alvi wkB2 and *G. apicola* wkB7 were inoculated from frozen glycerol stocks onto heart infusion agar supplemented with 5% sheep's blood, and incubated at 35°C in 5% CO_2. After 48 hours, bacteria were restreaked on fresh agar plates and grown for another 48 hours. Single colonies were harvested, passaged into Insectagro DS2 media (Corning Inc.), and grown at 35°C in 5% CO_2 for 24 hours.

RNA extraction

Bees were anesthetized by chilling at 4°C or by exposure to CO_2 and whole guts (midgut, ileum, and rectum) were removed. RNA from guts was extracted with 1 ml TRIzol (Life Technologies Corp.) following the manufacturer's RNA isolation protocol, with the addition of a one minute bead-beating step using ~0.5 ml 0.1-mm silica zirconia beads (BioSpec Products Inc.). Samples were resuspended in 50 µl of nuclease-free water, and then treated with Promega RQ1 RNase-Free DNase according to the manufacturer's protocol. RNA samples were quantified using a Qubit 2.0 with the Quant-iT RNA BR kit. cDNA was synthesized according to the Verso cDNA synthesis kit protocol (Thermo Fisher Scientific Inc.), using 0.5 µg of RNA per sample as template. cDNA samples were diluted 1:10 with nuclease-free water to be used as template for quantitative PCR.

16S rDNA microbial community analysis

DNA was extracted from dissected gut tissue as previously described [1]. DNA samples were resuspended in 50 µl nuclease-free water, quantified using the Quant-iT DNA BR kit, and amplified using primers specific for the V4 region of the bacterial 16S rRNA gene:

Hyb515F: 5'-TCGTCGGCAGCGTCAGATGTGTATAAGAGACAGGTGYCAGCMGCCGCGGTA-3'

Hyb806R: 5'-GTCTCGTGGGCTCGGAGATGTGTATAAGAGACAGGGACTACHVGGGTWTCTAAT-3'

Amplicons were sequenced on the Illumina Miseq 2 × 250 bp platform to assess gut microbiome composition. Reads were processed using QIIME v1.9.1 [2] as follows: raw reads were joined with the SeqPrep method using the parameters –g 0.06 and –n 0.2;

primer sequences were trimmed from joined reads, which were then quality filtered by applying cutoffs of phred > 29, max N = 0, and length between 230 bp and 270 bp (expected size ~250 bp); OTUs at 97% identity clustering were picked *de novo* using the default uclust algorithm. OTUs accounting for < 0.5% of reads for each sample were removed, as were OTUs without matches to 16S rDNA sequences or with matches to mitochondria or chloroplast. OTUs were assigned taxonomy by BLASTN searches against the Genbank nr database.

Quantitative PCR

qPCR was carried out on an Eppendorf Mastercycler ep realplex (*S. alvi* treatment experiment) or a Life Technologies ViiA 7 System (gut-fed treatment experiment). Ten-microliter reactions were set up in 96-well plates using 5 µl of iTaq Universal SYBR Green Supermix (Bio-Rad Inc.), 2 µl H_2O, 1 µl each of 5 µM forward and reverse primers, and 1 µl of 1:10 diluted cDNA template. The cycling conditions consisted of 95°C for 30 s; 10 cycles of 95°C for 5 s, 55°C for 15 s (-0.5°C per cycle), and 68°C for 20 s; 35–40 cycles of 95°C for 5 s, 50°C for 15 s, and 68°C for 20 s. A melting curve analysis was also conducted to ensure the PCR products were the correct sizes.

Detection of peptides in gut fluid and hemolymph

Newly eclosed bees were fed 5 µl of a single whole gut crushed in 500 µl 1:1 (w/v) sucrose-water. The guts were from adult hive bees (microbiota treatment) or newly eclosed bees lacking microbiota (control). After 5 days, hemolymph from the thorax and gut fluid from the rectum lumen were withdrawn using micropipettes after puncture by glass needles. Approximately 3 µl of each type of fluid was obtained per bee, and fluid was pooled from 5 –13 bees to obtain sufficient concentrations for analysis (~13–18 µl per sample). Samples were added to 100 µl of 0.1% (v/v) trifluoroacetic acid and boiled at 100°C for 10 min, then centrifuged at 15,000 g for 10 min. The supernatant was transferred to a new tube and boiled at 100°C for another 10 min, then spun at 15,000 g for 10 min. The supernatant was taken and frozen at -80°C until further enrichment of apidaecin by method of Danihlík *et al.* [3]. For peptide enrichment, Oasis WCX 1cc Vac Cartridges with 30 mg sorbent, 30 µm particle size (Waters Corp.), were conditioned with 1 ml methanol, then equilibrated with 1 ml HPLC-grade water. Samples were added to 900 µl 5% (v/v) formic acid and drawn through the sorbent via vacuum pressure. Cartridges were washed twice with 500 µl of 30 mM NH_4HCO_3 (pH 9), and then washed twice with 500 µl methanol. Peptides were subsequently eluted with 400 µl 50% (v/v) acetonitrile containing 5% (v/v) formic acid.

Prior to analysis, samples were concentrated by SpeedVac and resuspended in 12 µl 0.1% (v/v) formic acid. Samples were analyzed by reverse phase LC-MS/MS using a Dionex Ultimate 3000 Nano UPLC coupled to an Orbitrap Elite mass spectrometer (Thermo Fisher Scientific Inc.). The LC utilizes an Acclaim PepMap100 C18 Nano-Trap pre-column (75 µm × 2 cm, 3 µm particle size, 100 Å pore size) and an Acclaim PepMap Nano column (75 µm × 15 cm, 3 µm particle size, 100 Å pore size) for separation. Peptides were eluted from the column using a gradient of 0.1% (v/v) formic acid and water and 0.1% (v/v) formic acid and acetonitrile over 60 minutes. The Orbitrap Elite was operated using a targeted MS/MS method with precursor ions of 703.40 m/z, 665.70 m/z, 422.44 m/z, and 1054.59 m/z

being detected in the orbitrap at 60,000 resolution. Fragment ions were captured in the orbitrap and also detected at 60,000 resolution. Following acquisition, data were analyzed using Proteome Discoverer 1.4 (Thermo Fisher Scientific Inc.) with the SEQUEST HT search algorithm and target decoy method. Results reported are filtered for high (1% false discovery rate) confidence.

Bacterial challenge of bees

E. coli ATCC 25922, a strain commonly used for antimicrobial susceptibility assays, was grown in tryptic soy broth (BD Difco) at 37°C overnight to an OD_{600} of 0.5. Cells were centrifuged, washed with PBS, and then diluted to set concentrations (10^3–10^5 cells/μl) in PBS. Five days after inoculation with *S. alvi* or *G. apicola*, bees were chilled on ice in preparation for bacterial injection, and then 1 μl of PBS or *E. coli* was injected into the hemocoel laterally between the second and third tergite with a fine-tipped glass capillary. After infection, bees were returned to incubation at 34°C with sucrose-water and sterile pollen.

Measurement of E. coli survival in bee hemolymph

At 2 hours and 6 hours post-injection, hemolymph was collected from each bee and 0.5 μl aliquots of undiluted and 1:100 diluted hemolymph were plated onto tryptic soy agar. After overnight incubation at 37°C, the number of colony-forming units (CFUs) per agar plate was counted, multiplied by the dilution factor, and adjusted to reflect the number of surviving *E. coli* cells per μl of hemolymph.

Survival assays

We monitored and recorded the number of deceased bees in each cup cage approximately every 3 hours over 2 days. The initial number of bees in each cup was recorded at the time of bacterial injection. Survival curves were generated in Prism 6 (GraphPad Software Inc.). Multiple comparison tests were performed using the log-rank test.

G. apicola colonization trials

To test the effectiveness of gut colonization with *G. apicola*, preliminary trials were conducted with two *G. apicola* strains, wkB1 [4] and wkB7 [5]. Two methods of inoculation were used, hand-feeding and placing the cultured strains into the pollen food supply. Cultivation and feeding methods are as described in materials and methods. Sterile sucrose solution was used for the uninoculated controls. After 5 days, DNA was extracted from a subset of bees (Cohort 1) as previously described [1], and qPCR was performed using *Gilliamella*-specific primers as described in [6] to quantify colonization.

To determine if transmission of *G. apicola* directly from gut material improves colonization compared to using *in vitro* cultures, 5 μl of resuspended crushed guts from Cohort 1 bees were fed to a new set of newly eclosed workers. After 5 days, DNA was extracted from these bees (Cohort 2) and quantified by qPCR as above.

Supplementary Results

Microbiota composition of gut-fed bees

For the gut-fed qPCR experiment, two cohorts of the "full microbiota" treatment were used, each fed gut material from a single adult hive bee ("Gut A" and "Gut B"). For the uninoculated controls, bees were fed the gut material of a newly eclosed worker to control for the effect of ingesting gut material on gene expression. Microbiome composition was determined for 9 bees from each treatment, taken from the same cup-cages as bees kept for the qPCR experiment (three cup-cages for each of "Gut A", "Gut B", and "Control" groups; 3 bees taken from each cup-cage for DNA extraction/16S rDNA community analysis, 7–9 bees taken from each cup-cage for RNA extraction/qPCR analysis). Microbiome composition was also determined for the initial gut material used for inoculating "Gut A", "Gut B", and "Control" treatment groups.

Gut-fed "full microbiota" workers have microbiotas that largely resembled that of the source material (figure S1), although the relative proportions varied among individuals. Most individuals were successfully colonized with all members of the core microbiota (*S. alvi*, *G. apicola*, *F. perrara*, *Bifidobacterium* spp., and *Lactobacillus* Firm-4 and Firm-5). The inoculum for the control group had a low number of reads (290) compared to the "full microbiota" samples (average 23,731), suggesting that very few bacteria were present. The bees inoculated with the control gut material likewise lacked the characteristic bee gut microbial community (figure S1); however, some control individuals became colonized with *Lactobacillus* Firm-5. Although not all bees in this group may indeed be fully germ-free, reduced PCR amplification was observed for many control samples, suggesting a lower absolute bacterial load.

E. coli survival in hemolymph

Two of three trials showed a significant decrease of *E. coli* in the hemolymph of bees inoculated with *S. alvi* or *G. apicola* (figure 4). The third trial showed no difference; this trial was conducted on late-season bees (November 2015), whereas the other trials were on bees collected in the spring (May 2015). There may be seasonal immunological differences responsible for the observed differences between trials [7, 8], although this will need to be further tested.

G. apicola colonization trials

Up to 10^9 *Gilliamella* 16S rRNA gene copies were detected in bees monoinoculated with *G. apicola* strains. Colonization by strain wkB7 was more reliable than strain wkB1 (figure S5); however, hand-feeding of wkB7 resulted in lower colonization efficiencies than leaving cultured bacteria in the pollen food supply. We continued using hand-feeding in our survival trials, as it allowed for inoculation of a known quantity of bacteria per individual. Feeding of homogenized guts containing *G. apicola* resulted in better colonization than using *in vitro* cultures (figure S5, Cohort 2 treatments), but this method still did not allow for reliable colonization by strain wkB1. Based on these results, we decided to use strain wkB7 rather than wkB1 in our survival and *E. coli*-challenge experiments.

Supplementary References

1. Powell JE, Martinson VG, Urban-Mead K, Moran NA. 2014. Routes of acquisition of the gut microbiota of the honey bee *Apis mellifera*. *Appl. Environ. Microbiol*. 80(23):7378–7387. (doi:10.1128/AEM.01861-14)

2. Caporaso JG *et al*. 2010. QIIME allows analysis of high-throughput community sequencing data. *Nat. Methods.* 7(5):335–336. (doi:10.1038/nmeth.f.303)

3. Danihlík J, Šebela M, Petřivalský M, Lenobel R. 2014. A sensitive quantification of the peptide apidaecin 1 isoforms in single bee tissues using a weak cation exchange pre-separation and nanocapillary liquid chromatography coupled with mass spectrometry. *J. Chromatogr. A.* 1374:134–144. (doi:10.1016/j.chroma.2014.11.041)

4. Kwong WK, Moran NA. 2013. Cultivation and characterization of the gut symbionts of honey bees and bumble bees: description of *Snodgrassella alvi* gen. nov., sp. nov., a member of the family *Neisseriaceae* of the *Betaproteobacteria*, and *Gilliamella* apicola gen. nov., sp. nov., a member of *Orbaceae* fam. nov., *Orbales* ord. nov., a sister taxon to the order '*Enterobacteriales*' of the *Gammaproteobacteria*. *Int. J. Syst. Evol. Microbiol.* 63(6):2008–2018. (doi:10.1099/ijs.0.044875-0)

5. Zheng H, Nishida A, Kwong WK, Koch H, Engel P, Steele MI, Moran NA. 2016. Metabolism of toxic sugars by strains of the bee gut symbiont *Gilliamella apicola*. *MBio.* 7(6):e01326-16. (doi:10.1128/mBio.01326-16)

6. Martinson VG, Moy J, Moran NA. 2012. Establishment of characteristic gut bacteria during development of the honey bee worker. *Appl. Environ. Microbiol*. 78(8):2830–2840. (doi:10.1128/AEM.07810-11)

7. Steinmann N, Corona M, Neumann P, Dainat B. 2015. Overwintering is associated with reduced expression of immune genes and higher susceptibility to virus infection in honey bees. *PLoS ONE.* 10(6):e0129956. (doi:10.1371/journal.pone.0129956)

8. Gätschenberger H, Azzami K, Tautz J, Beier H. 2013. Antibacterial immune competence of honey bees (*Apis mellifera*) is adapted to different life stages and environmental risks. *PLoS ONE.* 8(6):e66415. (doi:10.1371/journal.pone.0066415)

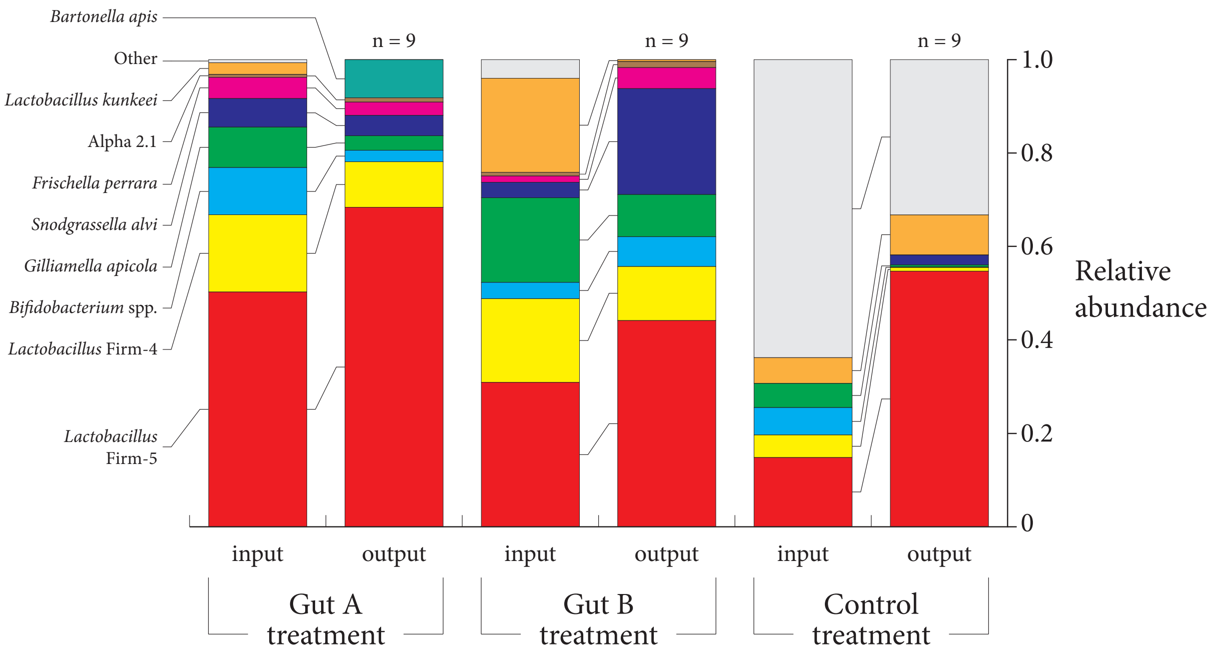

Figure S1. Gut microbiome composition of bees in the gut-fed qPCR experiment (figure 1). Bacterial taxonomic abundances for each individual bee were averaged to obtain relative proportions. Two cohorts of the "full microbiota" treatment were used, each fed gut material from a single adult hive bee ("Gut A" and "Gut B"). Input, the microbiome composition of the source gut. Output, the averaged microbiome from 9 individuals of treatment bees.

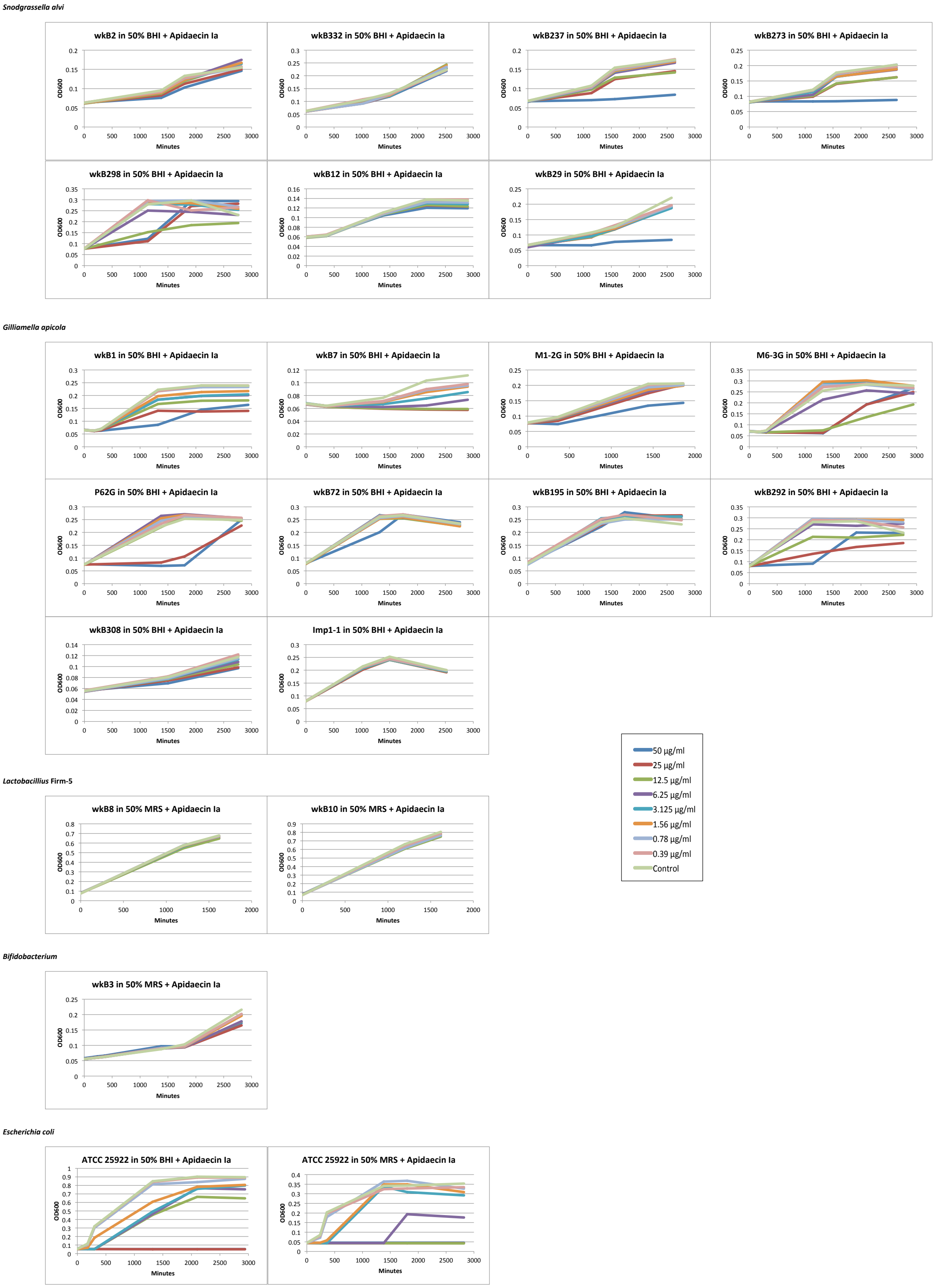

Figure S2. Apidaecin Ia resistance in the native bee gut bacteria. The minimum inhibitory concentration was determined in 1:1 diluted brain heart infusion broth (*E. coli*, *S. alvi*, *G. apicola*) or 1:1 diluted MRS broth (*E. coli*, *Lactobacillus*, *Bifidobacterium*).

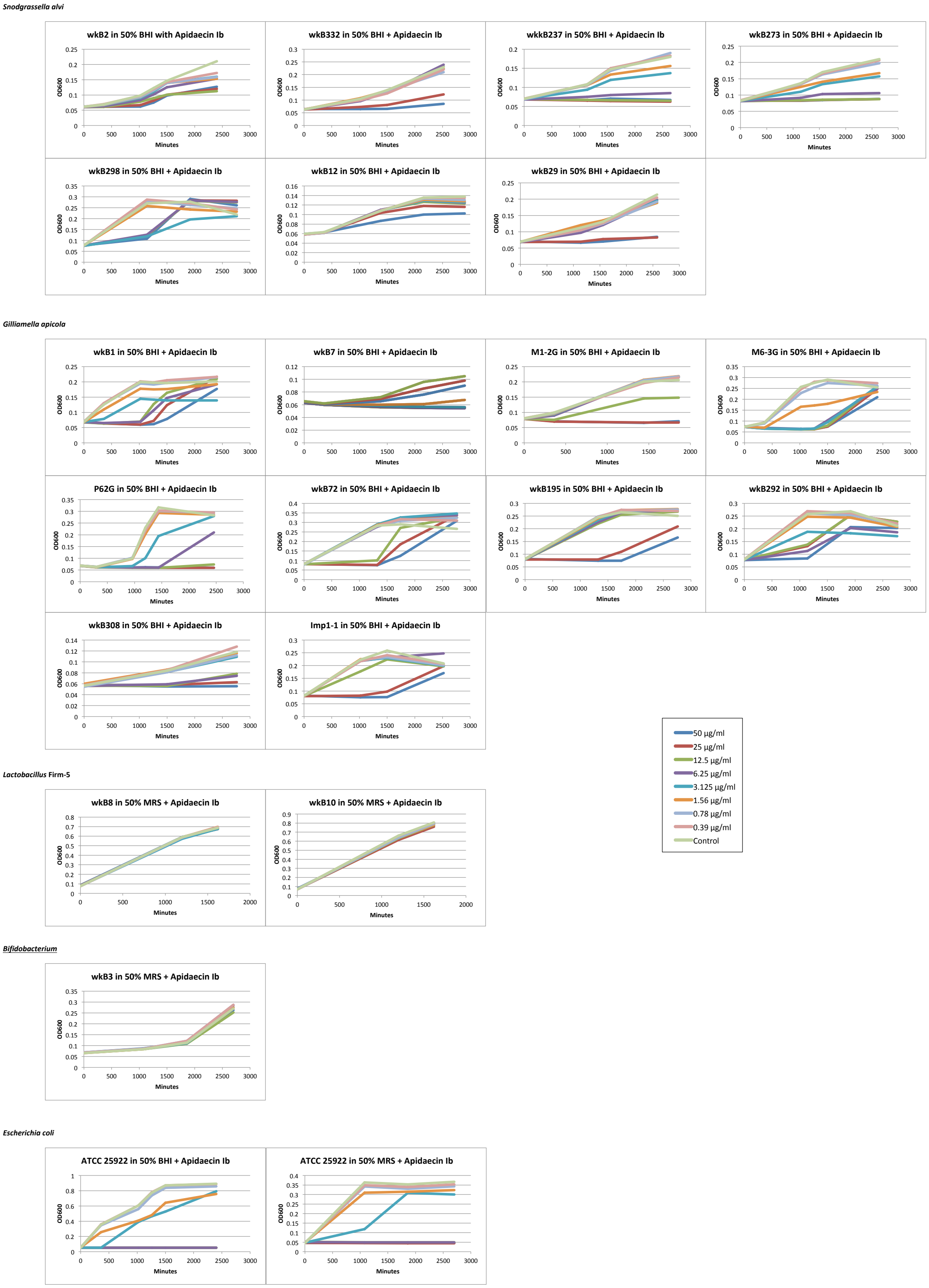

Figure S3. Apidaecin Ib resistance in the native bee gut bacteria. The minimum inhibitory concentration was determined in 1:1 diluted brain heart infusion broth (*E. coli*, *S. alvi*, *G. apicola*) or 1:1 diluted MRS broth (*E. coli*, *Lactobacillus*, *Bifidobacterium*).

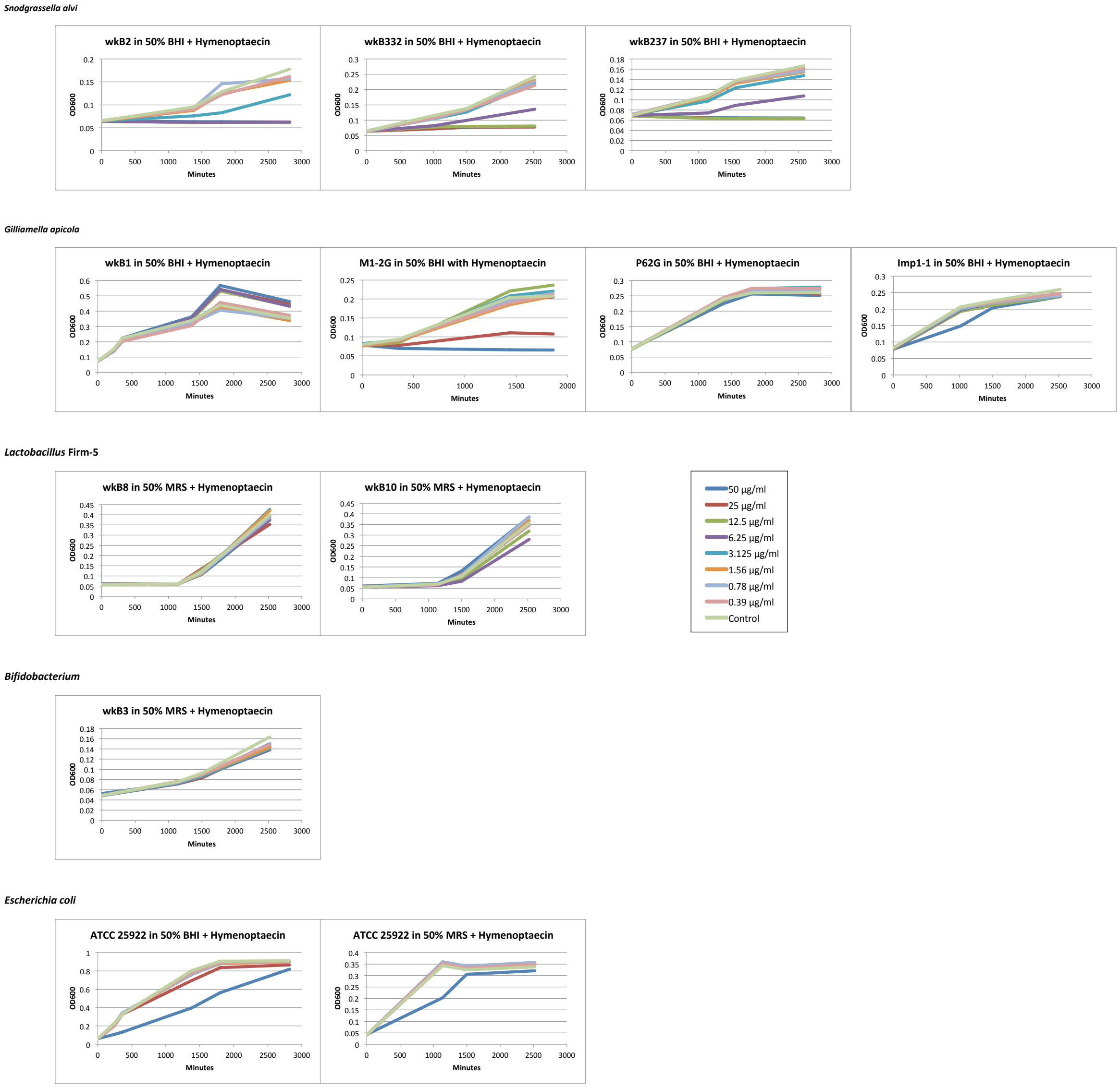

Figure S4. Hymenoptaecin resistance in the native bee gut bacteria. The minimum inhibitory concentration was determined in 1:1 diluted brain heart infusion broth (*E. coli*, *S. alvi*, *G. apicola*) or 1:1 diluted MRS broth (*E. coli*, *Lactobacillus*, *Bifidobacterium*).

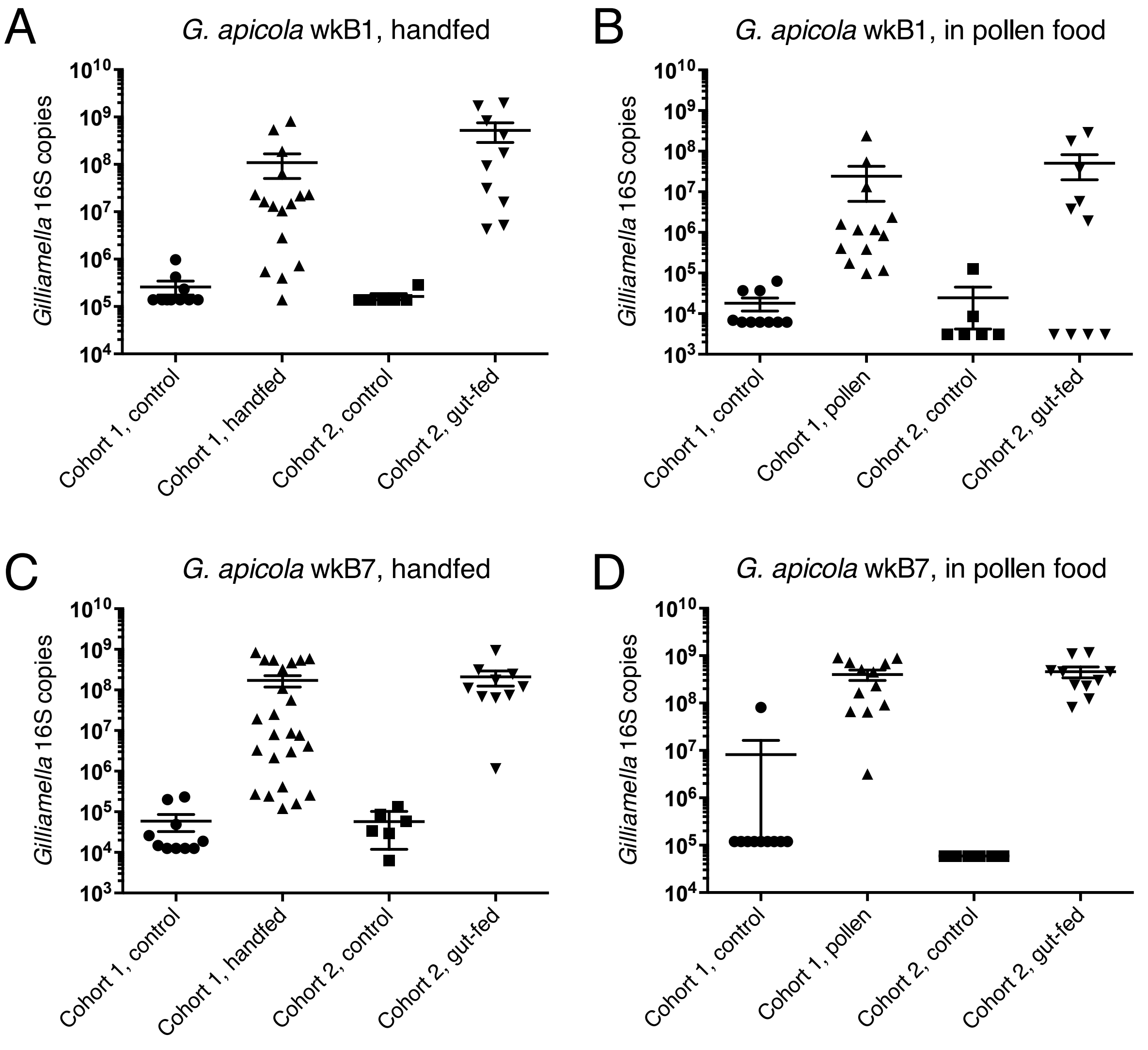

Figure S5. *G. apicola* colonization trials, qPCR results (A–D). 16S rRNA gene copy number represents copies per individual gut. Lowest values (e.g., in control treatments) reflect detection limit of qPCR assay, which differs due to run-to-run variation in the standard curve used for quantification.

Table S1. Antimicrobial peptide resistance of bacterial strains from *Apis* spp. and *Bombus* spp.

	**Apidaecin Ia**			**Apidaecin Ib**			**Hymen-optaecin**	
**Bacterial strain**	**MIC (μg/ml)**	**IZ (mm) 5 μg**	**IZ (mm) 10 μg**	**MIC (μg/ml)**	**IZ (mm) 5 μg**	**IZ (mm) 10 μg**	**MIC (μg/ml)**	**Host of origin**
Snodgrassella alvi								
wkB2	>50	-	-	12.5	-	-	3.125	*Apis mellifera*
wkB332	>50	-	-	25	-	-	6.25	*Apis mellifera*
wkB237	12.5	8	11	1.56	10	12	6.25	*Apis andreniformis*
wkB273	12.5	-	-	1.56	-	-	ND	*Apis florea*
wkB298	12.5	8	10	3.125	11	13	ND	*Apis cerana*
wkB12	>50	-	-	25	-	-	ND	*Bombus bimaculatus*
wkB29	50	-	-	25	-	-	ND	*Bombus vagans*
Gilliamella apicola								
wkB1	12.5	9	11	3.125	12	16	>50	*Apis mellifera*
wkB7	3.125	-	-	1.56	-	8	ND	*Apis mellifera*
M1-2G	50	-	8	12.5	9	11	25	*Apis mellifera*
M6-3G	12.5	11	13	1.56	14	16	ND	*Apis mellifera*
P62G	25	7	8	3.125	11	12	>50	*Apis mellifera*
wkB72	>50	-	-	12.5	9	11	ND	*Apis cerana*
wkB195	>50	-	11	25	9	11	ND	*Apis cerana*
wkB292	12.5	7	9	3.125	10	12	ND	*Apis cerana*
wkB308	25	-	-	6.25	8	10	ND	*Apis cerana*
Imp1-1	>50	-	-	25	8	10	>50	*Bombus impatiens*
Frischella perrara								
PEB0195	ND	8	9	ND	10	12	ND	*Apis mellifera*
***Lactobacillus* Firm-5**								
wkB8	>50	ND	ND	>50	ND	ND	>50	*Apis mellifera*
wkB10	>50	ND	ND	>50	ND	ND	>50	*Apis mellifera*
Bifidobacterium								
wkB3	>50	ND	ND	>50	ND	ND	>50	*Apis mellifera*
Escherichia coli								
ATCC 25922	1.56	8	12	1.56	9	11	50	

MIC, minimum inhibitory concentration in liquid media; IZ, inhibition zone diameter on solid media; -, no zone; ND, not determined. Growth curves from MIC assays are shown in figures S2–S4.
